# Discovery of a new sialic acid binding region that regulates Siglec-7

**DOI:** 10.1038/s41598-020-64887-4

**Published:** 2020-05-26

**Authors:** Nao Yamakawa, Yu Yasuda, Atsushi Yoshimura, Ami Goshima, Paul R. Crocker, Gérard Vergoten, Yuji Nishiura, Takashi Takahashi, Shinya Hanashima, Kana Matsumoto, Yoshiki Yamaguchi, Hiroshi Tanaka, Ken Kitajima, Chihiro Sato

**Affiliations:** 1 Biocience and Biotechnology Center, Nagoya University, Nagoya, 464-8601 Japan; 2 Department of Bioagricultural Sciences, Nagoya University, Nagoya, 464-8601 Japan; 3 Université de Lille, CNRS, UMR 8576, Unité de Glycobiologie Structurale et Fonctionnelle, Lille, 59000 France; 4 Division of Cell Signalling and Immunology, School of Life Sciences, University of Dundee, Dundee, DD1 5EH UK; 5 Department of Chemical Science and Engineering, School of Materials and Chemical Technology, Tokyo Institute of Technology, 2-12-1-H-101, Ookayama, Meguro, Tokyo, 152-8552 Japan; 6 Department of pharmacy, Yokohama University of Pharmacy, 601, Matana-cho, Totsuka-ku, Yokohama, Kanagawa 245-0066 Japan; 7 Structural Glycobiology Team, RIKEN Global Research Cluster, 2-1 Hirosawa, Wako, Saitama, 351-0198 Japan; 8 Present Address: Graduate School of Science, Osaka University, 1-1 Machikaneyama, Toyonaka, Osaka, 560-0043 Japan; 9 Present Address: Faculty of Pharmaceutical Sciences, Tohoku Medical and Pharmaceutical University, 4-4-1 Komatsushima, Aobaku, Sendai, Miyagi 981-8558 Japan

**Keywords:** Glycoconjugates, Glycobiology

## Abstract

Siglec-7 is a human CD33-like siglec, and is localised predominantly on human natural killer (NK) cells and monocytes. Siglec-7 is considered to function as an immunoreceptor in a sialic acid-dependent manner. However, the underlying mechanisms linking sialic acid-binding and function remain unknown. Here, to gain new insights into the ligand-binding properties of Siglec-7, we carried out *in silico* analysis and site-directed mutagenesis, and found a new sialic acid-binding region (site 2 containing R67) in addition to the well-known primary ligand-binding region (site 1 containing R124). This was supported by equilibrium dialysis, STD-NMR experiments, and inhibition analysis of GD3-binding toward Siglec-7 using synthetic sialoglycoconjugates and a comprehensive set of ganglioside-based glycoconjugates. Our results suggest that the two ligand-binding sites are potentially controlled by each other due to the flexible conformation of the C-C′ loop of Siglec-7.

## Introduction

Siglecs are sialic acid (Sia)-binding immunoglobulin-like lectins and the majority of them function as transmembrane receptors in the immune system via recognition of Sia residues^1^. The expression pattern of siglecs is often cell type-specific. Through cytosolic regions or adaptor proteins, they are able to regulate inhibitory or activating signals. Siglecs contain a Sia-binding N-terminal V-set domain followed by 1 to 16 C2-set Ig-domains that are considered to act as spacers or regulators of oligomerisation. Siglecs differ from most other mammalian Sia-binding lectins, such as selectins, with respect to the absolute requirement for Sia, but the details of their molecular interactions, including specific natural ligands and ligand-binding mechanisms, remain largely unclear. These are thought to be important for immune cell functions via Sia-binding because disrupting the interactions between Sias and siglecs can alter responses to infection, inflammation, autoimmunity, and cancer^1^. Therefore, understanding the role of ligand-binding in biological functions of siglecs is important.

The binding affinity between siglecs and naturally occurring sialylated glycoconjugates is known to be relatively weak (0.1–3 mM)^2^, and therefore biological interactions between siglecs and their ligands depend on clustering and multivalent binding. For example, mucins can display multiple ligands, and gangliosides can be clustered within lipid microdomains. To conquer the low affinity of Sia ligands for siglecs, recent research in Sia chemistry has focussed on the development of biochemical probes toward siglecs with high affinity and specificity^2–6^. Attempts to increase the affinity were successful, and in some cases materials were used for clinical therapy^7,8^. However, the precise details of how siglecs engage their ligands remain unclear.

Siglec-7 (CDw328) is the major siglec expressed by NK cells where it can function as an inhibitory receptor^4,9,10^. Siglec-7 consists of an extracellular N-terminal V-set Ig domain that is responsible for a Sia-binding, two C2-set Ig domains that act as spacers in projecting the ligand-binding site away from the membrane surface in the extracellular domain, an immunoreceptor tyrosine-based inhibition motif (ITIM), and an ITIM-like motif in the cytosolic region^9,10^. Based on the crystal structures, the V-set domain includes an essential Arg (R) 124 residue on the F β strand and a C-C′ loop structure, which are involved in Sia-binding at the primary binding site^11^. Siglec-7 has a preferred binding specificity towards the Neu5Acα2,8Neu5Acα- (diSia) sequence found in certain gangliosides such as the disialoganglioside, GD3, although certain branched α2,6-sialyl residues (DSGb5, DSLc4) are also known as preferred binding molecules^12,13^. In addition, the use of glycan arrays has demonstrated that Siglec-7 shows complex ligand specificity *in vitro*. So far, the natural ligands and the underlying recognition mechanisms in which Siglec-7 is involved are still unclear, although the high-affinity binding molecules that cluster diSia on the dextran have been developed^3^.

Cancer cells have been shown to be protected by Sia on their surface, and a hyper-sialylation state on the cancer cell surface may contribute to escape from NK cell killing^4,9^. Siglec-7 has recently been considered to be a target molecule for immunotherapy in cancer due to its potential role in Sia-dependent protection of carcinoma killing by NK cells^4^. Several attempts to regulate siglecs or target siglec-expressing cells have been made. For example, cancer cells were tagged with Sia epitopes with glycopolymers as probes to be targeted by the Siglec-7-expressing NK cells^4,7^. Besides the role in cancer, additional functions for Siglec-7 have been proposed. Expression of Siglec-7 has been reported to be reduced on NK cell subset (CD56^bright^) of obese humans^14^. Reduction of Siglec-7 via infection of hepatitis C virus led to dysfunctional NK cell phenotypes, and reduced degranulation and cytokine secretion^15^. In addition, decreased expression of Siglec-7 was also observed in patients with HIV infection^16^. Therefore, Siglec-7-expressing NK cells appear to be more functional, with enhanced activity, higher expression of several activation markers, and higher cytokine production than Siglec-7-negative NK cells^17^. Siglec-7 is also expressed by monocytes where antibody cross-linking can promote inflammatory responses^18^. Siglec-7 was shown to be present on a small subset of CD8^+^/CD3^+^ T cells in human blood. Using the Jurkat T cells as a model, overexpression of Siglec-7 was shown to inhibit the activation of T cells, and this function required R124 of Siglec-7^19^. Although Siglec-7 is involved in various biological functions, molecular details of ligand recognition and its potential regulation are still unclear.

To investigate the ligand regulating mechanisms of Siglec-7 in this study, we performed *in silico* docking analysis and found a new Sia-binding region that might be a regulator of the well-known binding site. We confirmed the presence of the new binding site with a mutated Siglec-7 using ELISA, equilibrium dialysis, STD-NMR, and inhibition analyses with synthetic sialoglycans. This information will prove valuable in the development of a novel effective molecule to regulate the functions of Siglec-7.

## Results and Discussion

### *In silico* molecular modelling analysis of Siglec-7

**Figure 1:**
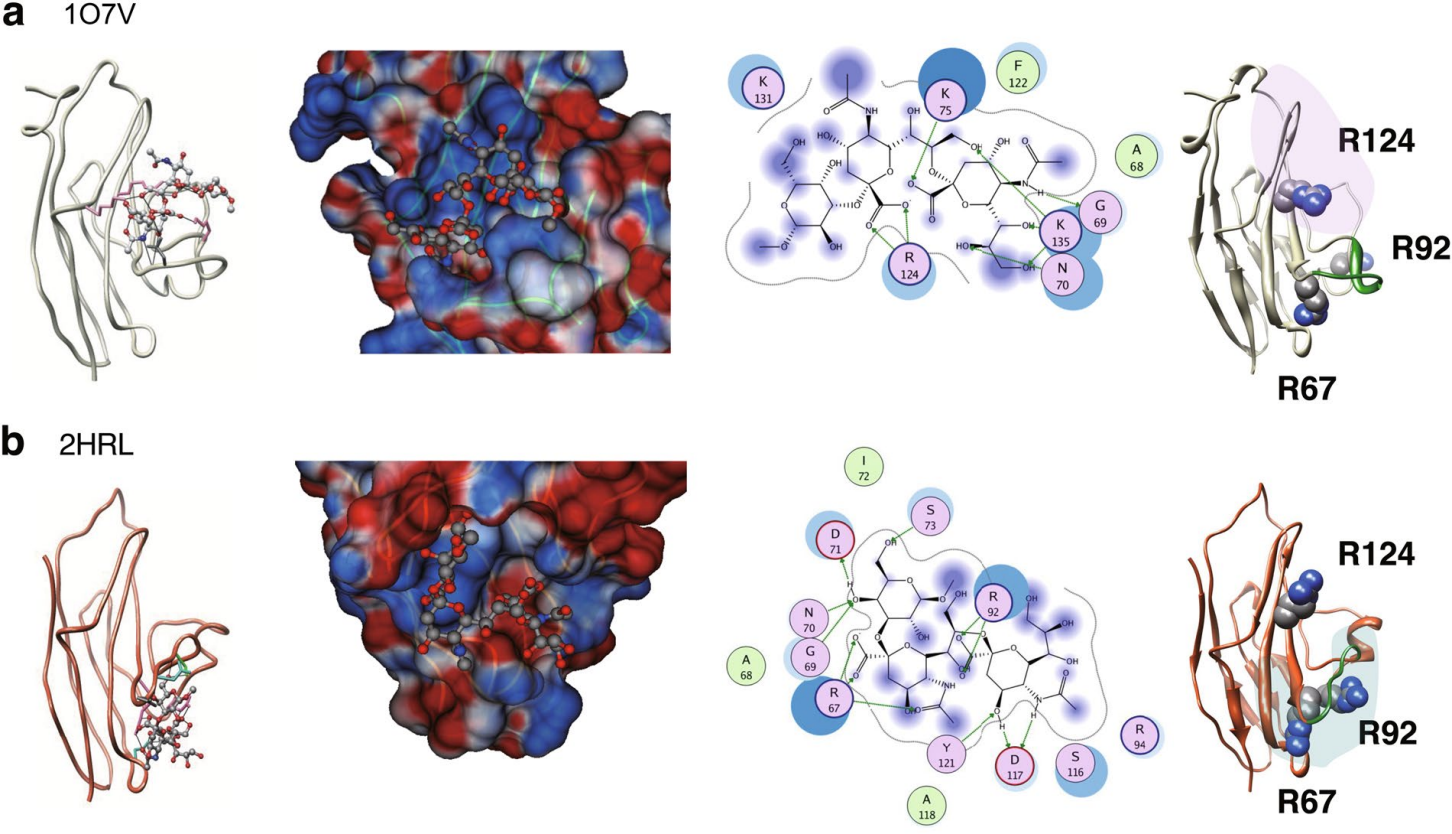
Docking results of Siglec-7 with diSiaGal. Docking with diSiaGal structure is depicted using 1O7V (**a**) and 2HRL (**b**) as templates. Left panels show the ribbon model of the Siglec-7V and diSiaGal structure. Middle left panels show the enlargement of the binding region. Blue and red indicate the basic and acidic regions of the protein. Middle right panels show the important amino acid residues for the glycan binding. The green arrow indicates the hydrogen bonding. The top hits are shown and the docking energy scores (U_*dock*_), the number of internal hydrogen bond and salt bridge, and amino acids and glycan residues involved in the hydrogen bond and salt bridge for 107 V and 2HRL were shown in Table 1. Right panel shows the amino acid residues that are predicted to be involved in the Sia-binding using docking analysis.

To date, sixteen human siglecs have been identified^1,2^ and structural information is widely available for the V-set ligand-binding domain. Indeed, for Siglec-7, three independent structures for each of the non-liganded (apo) (pdb code 1NKO, 1O7V, and 1O7S) and the liganded protein (pdb code 2G5R, 2DF3, and 2HRL) were previously solved^11,20–22^. The V-set domain shows an Ig-like fold based on a β-sandwich structure formed by two β-sheets consisting of strands A’GFCC’ and ABED (Supplementary Fig. 1a). We generated the superpositions by comparing these six PDB file structures (Supplementary Fig. 1b). The Siglec-7 crystal structures contained disordered regions, the flexible loops B-C and C-C′, and especially the C-C′ loop, which has been implicated in directing ligand-binding specificity^12,22^. However, little is known about how the flexible C-C′ loop regulates the binding preference of Siglec-7 to sialosides, and there is not enough structural information to interpret experimental data obtained to date. In the present study, we focussed on two different structures, the apo (1O7V)^11^ and the liganded forms (2HRL)^22^ that were applied in the docking experiment in Fig. 1. The striking difference between the two structures largely came from the C-C′ unique flexibility of the protein (Supplementary Fig. 1b). Actually, it has already been implicated that the conformational shift of C-C′ loop allows it to interact with the glycan core of the ganglioside^22^. In contrast, the ligand-binding site lying between strands A and G did not represent a large conformational change in the ribbon structure. However, the local changes revealed in the side chain of R124, K131, and K135 compared to other proximal amino acid residues, Y26, N133, and W132, frequently occurred as shown in the superpositions of all six Siglec-7 structures (Supplementary Fig. 1c). Indeed, it has been demonstrated that the side-chain of K131 masks the guanidinium group of R124 to obscure the ligand-binding when in the apo-structure, and that this residue moves away to reveal the primary arginine residue, which allows it to interact with the carboxyl group of Sia^22^. It is therefore of interest to study in detail the effect of local shape modifications on the Siglec-7 structure for the sialoside docking.

It has been shown that siglecs exhibit a preference for the glycosidic linkage of Sia to adjacent carbohydrates^1^. Among all siglecs, Siglec-7 shows a striking preference for α2,8-linked diSia and for Sia with an α2,6-linkage to an internal *N*-acetylglucosamine^12,13,23^. The α2,8-linked diSia structure is frequently presented in b-series gangliosides such as GD3 and GT1b, and the biological relationship between b-series ganglioside and Siglec-7 has been reported^24,25^. To examine whether both large conformational changes in flexible loop and local shape modifications on essential binding site of Siglec-7 structure alter its specific ligand recognition, we adopted an approach involving the use of computer-aided molecular model to examine the nature of Sia-binding on Siglec-7. To test the binding potential toward the Sia recognition site of Siglec-7 V-set domain (Siglec-7V), we docked Neu5Acα2,8Neu5Acα2,3 Gal (diSiaGal) structure that contains the peripheral GD3 glycan structure (Supplementary Fig. 2), which is considered as a ligand of Siglec-7V.

### Docking simulation of disialylated glycans with two comparable models of Siglec-7V (1O7V and 2HRL)

DOCK 6.1 was used here to investigate the docking state of the diSiaGal with Siglec-7V models whose *N*-linked oligosaccharide on the N105 was excluded to simplify the receptor structure (1O7V and 2HRL). Several kinds of clusters were generated as docking sites in each model, which nearly covered the entire surface area of protein structure. The ligand interactions of 1O7V and 2HRL are shown in Table 1. The column named “docking site” represents the site to which diSiaGal was bound and formed a relatively stable complex in each Siglec-7V compared to that in other docking sites. In addition, the list of hydrogen bond and salt-bridge contacts of the complex models are also presented. The ligand interactions listed in the table are represented in Fig. 1a for 1O7V and Fig. 1b for 2HRL. When 1O7V was used as a structural template, the diSiaGal-1O7V complex achieved relatively low binding energy at less than −241 kcal/mol, representing the energy term in U_*dock*_ (kcal/mol), which comprises the van der Waals and electrostatic interactions between Siglec-7V and sialoside, suggesting that the disialylated glycan/1O7V complex is stable. As shown in the detailed interaction of diSiaGal and 1O7V docking region in the putative Sia-binding region (site 1) (Fig. 1a), the complex showed two rigid key salt bridges between R124 and carboxyl group of Sia, indicating that R124 is a key amino acid for Sia-binding, which was reported previously, and is successfully predicted by this simulation procedure. It is noted that the non-reducing terminal Sia residue on GT1b interacted with R124 in the crystal structure, while the penultimate Sia residue interacted with R124 in the docking results. However, this may be a subtle difference because the carboxyl groups of the non-reducing terminal and penultimate Sia residues are actually very close, and because the diSia structure used in the docking experiments is more flexible than that in GT1b due to the absence of the GM1b core structure. From the results of 2HRL docking, no sialoside complex on the putative binding site (site 1) with less than −200 kcal/mol of binding energy was found but one model was observed (U_*dock*_, −151). Notably, instead, the lowest binding energy complex of diSiaGal (U_*dock*_, −165) was discovered in an unexpected site (site 2) that is localised in a cavity placed under the C-C′ loop (Fig. 1b). We also docked the same ligand to 2G5R and 2DF3 and found the docking models at the site 2 with low binding energy (Supplementary Table 1). Based on the comparable ribbon diagram (Fig. 1b and Supplementary Fig. 1b), the C-C′ loop of 2HRL were remarkably shifted and rearranged compared to 1O7V, while no distinct difference was observed in the strands A, F, and G where the sialoside docked in 1O7V. As shown in the detailed interaction of diSiaGal and 2HRL in the newly found Sia-binding site (site 2) (Fig. 1b), the complex showed rigid key salt bridges between R67 and the carboxyl group of Sia (Sia1) and R92 and the carboxyl group of Sia (Sia2) (Table 1), indicating that R67 and R92 might be key amino acid residues for Sia-binding.

**Table 1:** Docking results for the low-binding-energy complexes of a disialylated glycan to 1O7V and 2HRL models using DOCK 6.1.

Model protein	1O7V^a^	1O7V	2HRL	2HRL^a^
Docking site	Site 1	Site2	Site 1	Site 2
U_*dock*_ (kcal/mol)	−241	n.d.^b^	−151	−165
The number of internal hydrogen bond and salt bridge	8		5	10
**Amino acid and glycan residues involved in the hydrogen bond and salt bridge**				
Y64			Sia2	
R67				Sia1,Sia2
G69	Sia2			Gal
N70	Sia2			Gal
D71				Gal
K75	Sia2			
R92				Sia2
R94				Sia2
S116				Sia2
R124	Sia1		Sia1	
E126			Sia1	
G128			Sia2	
K131			Sia2	
N133			Gal	
K135	Sia2			

Combinations of amino acid residues and glycan residues that are involved in hydrogen bonding and salt bridge are listed. Sia 1, Sia 2, and Gal stand for residues in Sia2-Sia1-Gal, corresponding to Neu5Acα2-8Neu5Acα2-3Gal-Me. The number of intermolecular hydrogen bond and salt bridge, the binding energy (U_*dock*_) are also shown in the list. These are top-hit conformations, and two of them are shown in Fig. 1.

^a^The model was shown in Fig. 1. ^b^n.d. not detected.

### *In vitro* ligand-binding activity of Siglec-7

**Figure 2:**
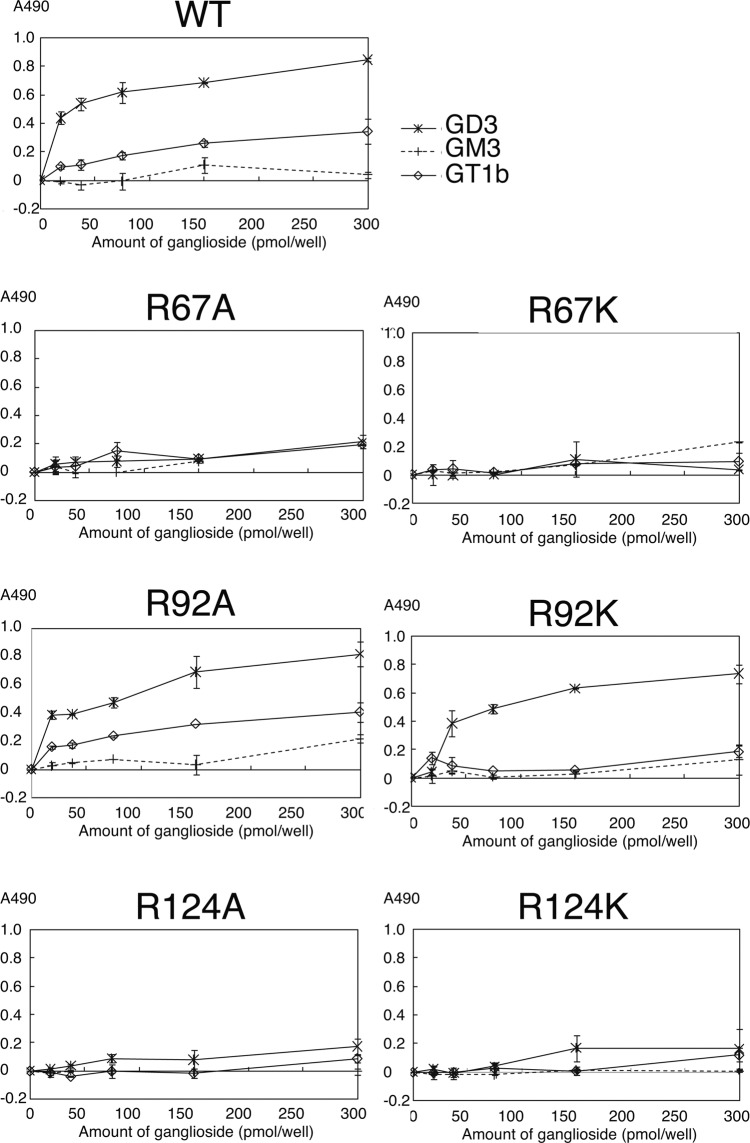
The binding of Siglec-7 toward gangliosides. Siglec-7EcFc were analysed for binding to gangliosides, GD3, GM3, and GT1b using ELISA. The gangliosides were coated onto a plastic well plate (0–300 pmol/well) and blocked. After washing, the premixed Siglec-7EcFc with anti-human Ig antibody labelled with POD were overlaid and incubated. The binding of Siglec-7EcFc was measured after colorimetric development. WT, wild type, R67A, R67K, R92A, R92K, R124A, and R124K mutants were used.

**Figure 3:**
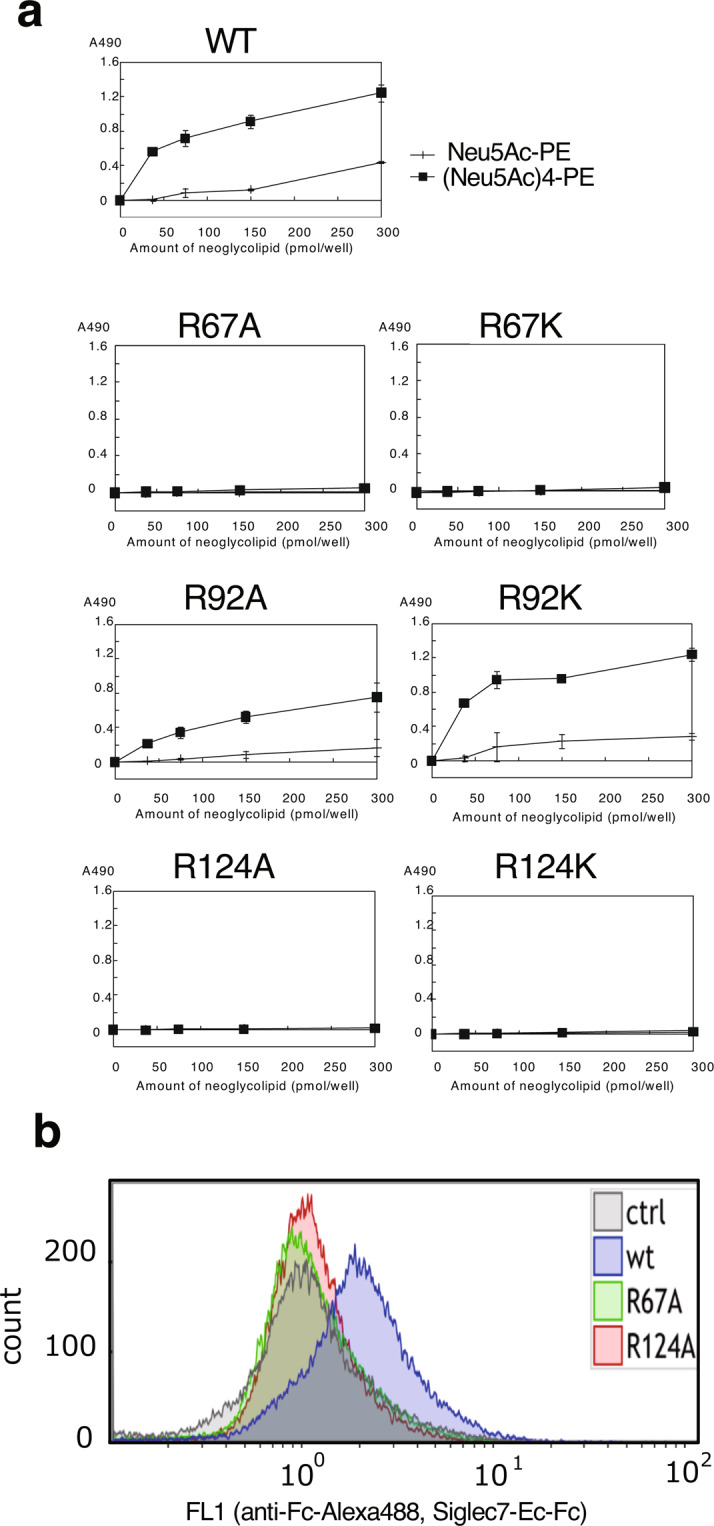
The binding of Siglec-7 toward α2,8-triSia. The triSia binding of Siglec-7EcFc was analysed using ELISA. (Neu5Ac)_4_-PE (triSia) and (Neu5Ac)-PE (negative control), the phosphatidylethanolamine (PE)-conjugate with tetraSia and monoSia, respectively, were coated onto the plastic wells (0–300 pmol/well) and blocked. After washing, the premixed Siglec-7EcFc with anti-human Ig antibody labelled with POD were incubated. The binding of Siglec-7EcFc was measured after colorimetric development. WT, wild type, R67A, R67K, R92A, R92K, R124A, and R124K mutants were used. (**b**) Binding of cell surface Sia of mouse macrophage RAW using flow cytometry. The RAW cells were incubated with Siglec-7EcFc (WT), Siglec-7EcFc (R67A), and Siglec-7EcFc (124A). After washing, Alexa 488-labelled anti-human Ig were incubated. Then the cells were analysed for Siglec-7EcFc binding using flow cytometry.

To confirm that Siglec-7 has a second Sia-binding region (site 2) and that R67 and R92 are involved in the Sia-binding, we focussed on the basic amino acids that are considered to be involved in the Sia-binding by docking simulations. In site 1, R124 has been already reported to be important for Sia-binding^1^. In site 2, we focussed on the two basic amino acids R67 and R92 because R67 and R92 were shown to form salt bridges with internal Sia1 and terminal Sia2, respectively, based on the docking analysis with 2HRL (Fig. 1 and Table 1). Therefore, we prepared soluble Siglec-7EcFc (WT) and mutated Siglec-7EcFc whose amino acids were mutated as R67A, R67K, R92A, R92K, R124A, and R124K. We prepared each Siglec-7EcFc (Supplementary Fig. 3a) and analysed their conformation using three anti-Siglec-7 antibodies (Supplementary Fig. 3b). All these Siglec-7EcFc mutants showed the same activity toward these Siglec-7 specific antibodies, indicating that these proteins had almost the same conformation as evaluated by the antibodies. We then analysed their ligand-binding activity toward GD3, GM3, and GT1b. Among them, GD3 was shown to be a ligand for Siglec-7EcFc via site 1 using cellular analysis^9^ and GT1b binding was confirmed by the co-crystalised structure of 2HRL. As shown in Fig. 2, WT showed higher binding activity toward diSia containing ligand GD3, and almost half that activity toward GT1b. Almost no binding activity was observed toward GM3. All these data are consistent with previous results^3^ where 124A and 124K showed no ligand-binding activity, and it was again shown that R124 in site 1 is important for diSia binding^1^. Interestingly, R67A and R67K also showed significantly lower binding activity toward GD3 and GT1b although the site 1 at R124 was intact. R92A and R92K showed almost the same binding activity toward GD3. Therefore, it is clearly shown that R67, which is located far from the R124 in site 1, and predicted to be a new binding site by docking methods, is important for diSia binding. It is also consistent that R67 is the only amino acid that is involved in binding by docking results using 2G5R and 2DF3 (Supplementary Table 1). Next, we analysed the binding activity toward di/oligosialic acid (di/oligoSia) using (Neu5Ac)_n_-PE by ELISA. The reducing terminal end of di/oligoSia is open, linked to PE, and present as a spacer, therefore, the intact degree of polymerisation (DP) is considered to be n-1^26^. Before the experiments, we prepared sialidase-treated sample of each Siglec-7EcFc (Supplementary Fig. 3b), and analysed the binding. We found that no significant difference was observed (Supplementary Fig. 3d), indicating that the purified Siglec-7EcFc can be used without sialidase treatment like intact Siglec-7. We analysed the binding activity of mutated Siglec-7EcFc toward (Neu5Ac)_3_ ((Neu5Ac)_4_-PE) and found that not only R124A/K, but also R67A/K showed no activity (Fig. 3a). Consistent with the results in Fig. 2, R92A and R92K showed binding toward (Neu5Ac)_3_; however, the binding of R92A toward triSia was half that of the WT. To confirm that Siglec-7EcFc can bind to the cell surface ligand, we used mouse macrophage RAW264.7 cells (Fig. 3b). We analysed the di/oligo/polySia structure of the RAW cell surface using anti-di/oligo/polySia specific antibodies^27^ and the cells were clearly shown to possess diSia and triSia on their surface (Supplementary Fig. 4). Then, we analysed the Siglec-7EcFc binding using flow cytometry, toward diSia or triSia structures, which are considered to be ligands for Siglec-7, and found that only WT showed a strong binding toward cell surface ligands; R67A showed slight binding and R124A showed no binding. This clearly indicated that not only R124 in site 1, but also R67 in site 2 are involved in the cell surface binding (Fig. 3b).

### Estimation of binding pockets of Siglec-7 for diSia structure

It was suggested that Siglec-7 has a second Sia-binding site by docking analysis (Fig. 1), and it was shown that the binding activity of R67A, whose binding site around R124 is considered to be intact, toward the diSia structure was drastically decreased compared with that of WT (Fig. 2). Thus, it is important to understand the number of the ligand-binding sites of Siglec-7 for the diSia structure together with their binding property. For these purposes, we performed an equilibrium dialysis experiment using Siglec-7-EcFc and rhodamine green (RG)-conjugated Neu5Acα2,8Neu5Acα2,3-Gal in solution (Supplementary Fig. 5). The dose-dependent binding profiles of the ligands and the Scatchard plots are shown for WT (Supplementary Fig. 5a,b) and for R67A (Supplementary Fig. 5c,d). Based on the results of the Scatchard plots, the apparent *K*A and the number of the ligand-binding sites (n) were calculated (Supplementary Table 3). WT showed binding toward Neu5Acα2,8Neu5Acα2,3-Gal-RG with *K*_A_ 6.0 × 10^8^ ± 2.1 × 10^8^ M^−1^ and n = 4.3 ± 0.81. For R67A, the binding was unexpectedly observed toward Neu5Acα2,8Neu5Acα2,3-Gal-RG with *K*_A_ 3.8 × 10^8^ ± 2.1 × 10^8^ M^−1^ and n = 3.0 ± 0.080. These results suggest that there are 4.3 and 3.0 binding sites with similar *K*_A_ values in WT and R67A, respectively. However, unfortunately, the simple Scatchard-plot analysis is intrinsically unsuitable for obtaining precise parameters for multiple binding site-model with similar *K*_A_ values. Therefore, we could not adopt these parameters as conclusive ones. It should rather be noted that our equilibration dialysis data for Siglec-7 WT and R67A would not follow a single binding site-model, which is conventionally accepted. Thus, these results indirectly indicate that there must be more than one binding site on the Siglec-7 molecule. Interestingly, R67A did not effectively bind to GD3 glycosphingolipid, which immobilized on the ELISA plate (Fig. 2), irrespective of the presence of intact site 1 in R67A. Thus, the site 1 of R67A appeared not to have access to the diSia structure in the solid-phase ligand, probably due to an induction of conformational change of the C-C′ loop. Unlike R67A, R124A showed no stable binding to the diSia structure in solution (Supplementary Fig. 5e,f), although site 2 around R67 should remain intact in R124A. We performed experiments several times to obtain the reproduced data (see new Supplementary Table 3). We do not know the reason for the unstable results for R124A exactly; however, one of the possibilities is that the R to A alteration might induce conformational changes of the C-C′ loop to close or shield site 2.

**Figure 4:**
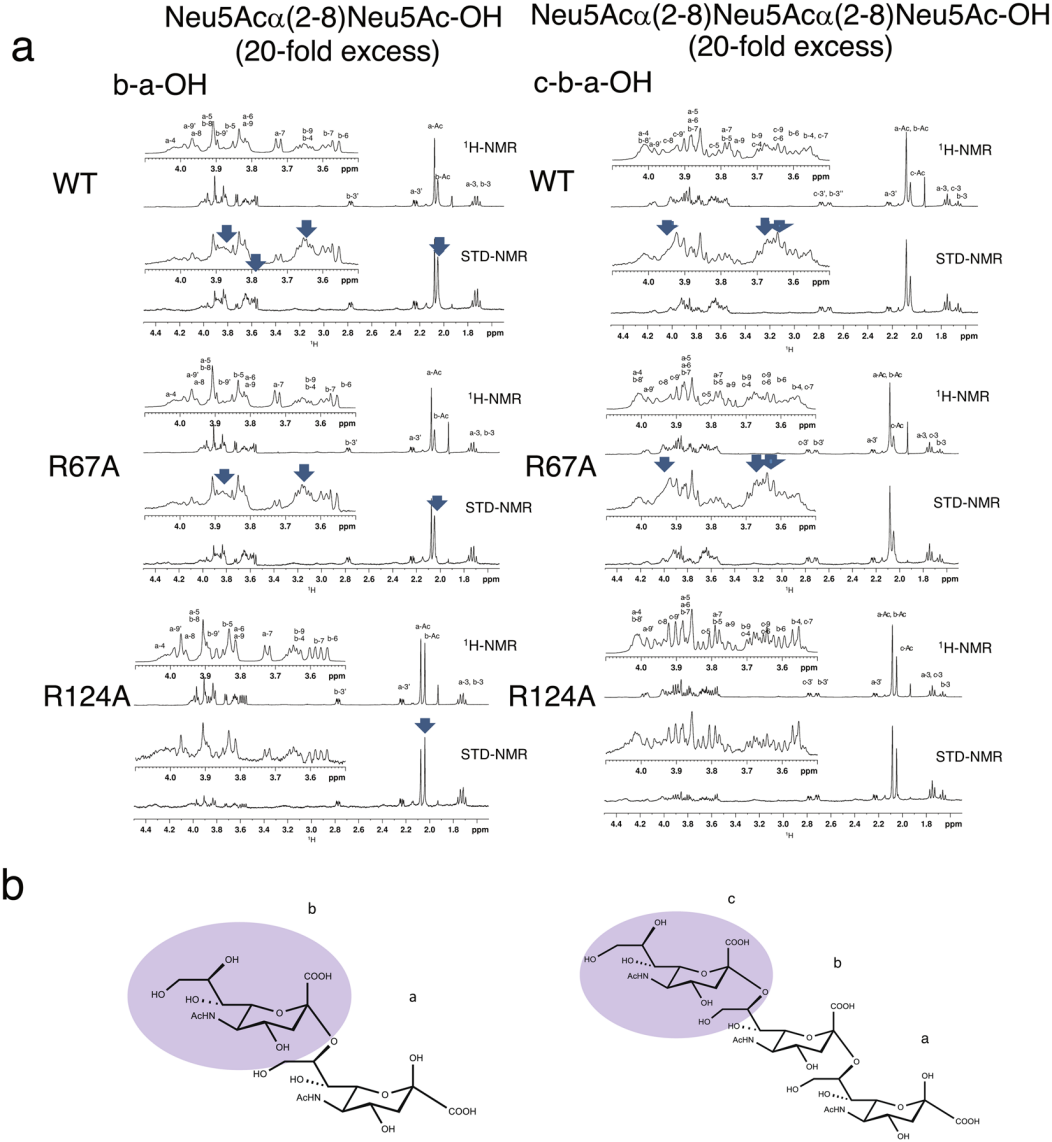
STD-NMR of Siglec-7 using α2,8-diSia and α2,8-triSia. STD-NMR spectra of Neu5Acα(2-8)Neu5Ac-OH (diSia) or Neu5Acα(2-8)Neu5Acα(2-8)Neu5Ac-OH (triSia) in the presence of Siglec-7V WT and its mutants, R124A and R67A, with a molar ratio of 20:1. (**a**) Upper panels; reference ^1^H- NMR spectra irradiated at 40 ppm and its expansion of 4.1–3.5 ppm. Lower panel; STD spectra obtained by subtraction of the saturation spectra irradiated at 0.15 ppm from the reference spectra, and its expansion from 4.1–3.5 ppm. The initial protein concentration was 55 μM in 10 mM PBS (pH 7.9). The probe temperature was set at 10 °C. The arrows indicate the signals receiving significant saturation effect. (**b**) DiSia and triSia structure. The area that is involved in the binding is shown in purple.

To gain more insight into the ligand-binding property of Siglec-7 in solution, we then performed STD-NMR^28^ of Siglec-7V domain using both diSia and triSia structures as ligands. Through the analyses, we could map the binding faces of the ligands if they interacted. In addition, we could also predict the interaction of the protein with the ligands by observing changes of chemical shifts of signals at the aromatic/amide region (7.7–6.2 ppm) and aliphatic region (1.3–0 ppm). When the STD-NMR spectra of diSia and triSia were compared with their ^1^H-NMR spectra, signals for the non-reducing terminal residues of diSia (b residue) and triSia (c residue) were changed in the spectra of WT (Fig. 4a, WT arrows) and R67A (Fig. 4, R67A arrows). In contrast, very little changes of those signals were observed in that of R124A (Fig. 4a, R124A). It is thus indicated that non-reducing terminal residues of diSia and triSia structures are involved in binding to the WT and R67A, and that very little or no interactions happened between the diSia and triSia structures and R124A. The titration experiments with diSia also showed that diSia bound to WT and R67A in solution because chemical shifts of aromatic protons around 6.2–6.5 were changed (Supplementary Fig. 6, WT 6.2–6.5 ppm), although the chemical shift profiles for protons around 6.7–7.2 ppm were different between WT and R67A. In the case of the titration with triSia, the chemical shifts of signals at 7.2–7.4 and 6.4 changed when using WT and R67A (Supplementary Fig. 6). The significant but very small change of chemical shift of signal at 6.4 ppm was only observed in the case of diSia for R124A. These results indicate that the diSia was obviously recognised by WT and R67A in solution. The binding toward diSia observed in STD-NMR is consistent with the results obtained using equilibrium dialysis experiments (Supplementary Fig. 5).

The solid-phase experiments revealed that both R124A and R67A did not bind to diSia or triSia glycosphingolipids (Figs. 2 and 3). In contrast, in solution, R67A, but not R124A, was capable of binding to diSia-glycan. Taken together, it is strongly suggested that the change of R at 124 to A influenced not only site 1, but also secondary binding site (site 2) involving R67, probably due to conformational changes of the C-C′ loop. It is also suggested that lipid components might affect the orientation of the C-C′ loop. For siglec-7-GD3 binding, it is noteworthy that the ceramide composition of GD3 is very important for Siglec-7-GD3 binding^29^. The hydroxyl ceramide of GD3, which is excluded from lipid raft cannot be recognised by Siglec-7, indicating that clustering of GD3 (glycan) and/or lipid-region are important for Siglec-7 recognition mechanism.

### Inhibitory activity of synthetic sialosides toward Siglec-7-GD3 binding

**Figure 5:**
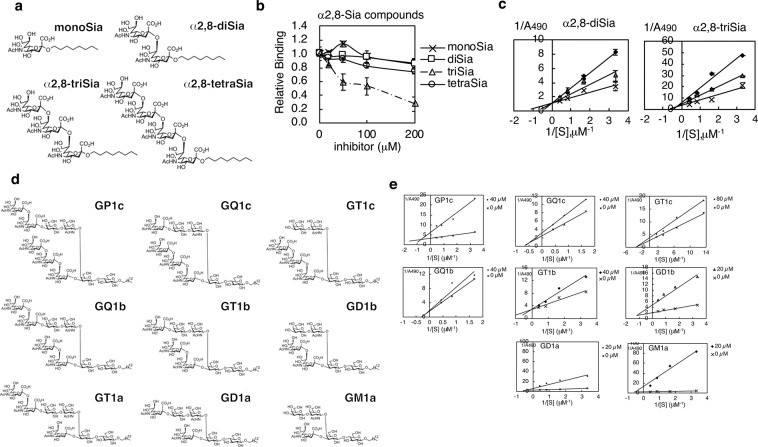
Inhibition analysis of chemically synthesised sialyl glycans toward GD3-Siglec-7EcFc binding. (**a**) α2,8-Sia compounds used in this study. The octyl glycosides of monoSia, α2,8-diSia, α2,8-triSia, and α2,8-tetraSia were chemically synthesised. **(b**) Inhibition effect of the α2,8-Sia compounds on the GD3-Siglec-7EcFc interaction. The plate was coated with GD3 (300 nM). The complex mixture with Siglec-7EcFc and anti-human IgG+IgM+IgA was incubated with or without α2,8-Sia compounds (final concentrations of 0–200 μM). This solution was then added to the GD3-immobilised plate, and the bound Siglec-7EcFc to the GD3 plate was measured as described under experimental procedures. The bound Siglec-7EcFc on GD3 without α2,8-Sia compounds was set to 1.0. The experiments were performed three times and SDs are shown. (**c**) Double reciprocal plots for the Siglec-7-GD3 interaction in the presence and absence of the α2,8-Sia compounds. The plots in the absence of α2,8-Sia compounds (cross, 0 μM) and those in the presence of the compounds (triangle, 200 μM; circle, 400 μM) are shown. X-axis shows the reciprocal concentration of GD3, 1/[S] (μM^−1^). Y-axis shows the reciprocal plot of the absorbance A490 obtained from ELISA analysis. The plots show the average and SD of three experiments. The y-axis intercepts in the left and right panels show different 1/A490 values from each other; however, the data for each panel were taken on the same plate on the same day. Thus, the type of inhibition can be evaluated based on the shapes of curve in either of the graphs. (**d)** Tetradecyl-ganglioside glycans used in this study. These compounds were chemically synthesised. a-series, GM1a, GD1a, and GT1a; b-series, GD1b, GT1b, and GQ1b; c-series, GT1c, GQ1c, and GP1c. (**e**) Double reciprocal plots for the Siglec-7-GD3 interaction in the presence and absence of tetradecyl-ganglioside glycans. The plots in the absence of tetradecyl ganglioside glycans (cross, 0 μM) and those in the presence of the glycans (triangle, 40 μM) are shown. X-axis shows the reciprocal concentration of GD3, 1/[S] (μM^−1^). Y-axis shows the reciprocal plot of the absorbance A490 obtained from ELISA analysis (shown in Supplementary Fig. 7a). The plots show the average of the three experiments. The experiments were carefully performed as described in (**c**).

The binding experiments of Siglec-7 and the mutants with the immobilized glycolipids (Figs. 2 and 3) and free glycan ligands (Supplementary Fig. 5 and Fig. 4) concluded that site 2 is present on Siglec-7 in addition to site 1. To further confirm this conclusion, we performed inhibition analysis using a variety of Sia conjugates. We prepared synthetic sialoglycomaterials, i.e. octyl glycosides of monoSia, α2,8-diSia, α2,8-triSia, and α2,8-tetraSia^30,31^ (Fig. 5a), and evaluated them as inhibitors for the Siglec-7EcFc-GD3 (α2,8-diSia epitope on ganglioside) interaction. Of those α2,8-compounds, only α2,8-triSia showed lower IC_50_ value (<100 μM) (Fig. 5b). The α2,8-diSia, the same epitope in GD3, showed an inhibition activity; however, the effect was weak. Notably, we recently demonstrated that diSia units-containing glycopolymers, such as poly-diSia compound^6^ and diSia-dextran^3^, showed extremely higher inhibition effects on the GD3-Siglec-7EcFc interaction than diSia. These results indicate that clustering of diSia is important for the α2,8-diSia-mediated inhibition of the GD3-Siglec-7 binding.

To determine the inhibition type of α2,8-diSia and α2,8-triSia, the double reciprocal plots were analysed because the inhibition type tells if another binding site other than the primary ligand-binding site is present. Competitive inhibition would show that inhibitors compete the same binding site with the ligand. If the double reciprocal plots with and without an inhibitor shared the same y-intercept values, the inhibitor is considered to bind to the same binding pocket as the ligand GD3. If the double reciprocal plots with and without an inhibitor had the same X-intercept value, the inhibitor is a non-competitive inhibitor that is considered to bind to the different site than that for the ligand GD3. As predicted, the α2,8-diSia showed competitive inhibition (Fig. 5c, α2,8-diSia). On the other hand, the α2,8-triSia showed non-competitive inhibition (Fig. 5c, α2,8-triSia). Thus, the binding of Siglec-7 to GD3 was inhibited by α2,8-diSia and α2,8-triSia; however, the inhibition mechanism was not the same. A binding site for α2,8-diSia structure can be directly competed by α2,8-diSia-containing materials, while it can be indirectly inhibited by α2,8-triSia through its binding to another allosteric site on Siglec-7. Taken together, it was indicated that Siglec-7EcFc has at least two binding pockets.

To further investigate the inhibitory effects of the ganglioside glycans on GD3-Siglec-7 binding, we chemically synthesised comprehensive ganglioside glycans, GP1c, GQ1c, GT1c, GQ1b, GT1b, GD1b, GT1a, GD1a, and GM1a (Fig. 5d). Using these tetradecyl-compounds as inhibitors, we analysed their effects on GD3-Siglec-7EcFc interaction (Supplementary Fig. 7a). The *K*_i_ values for these compounds are summarised in Table 2. We also analysed the double reciprocal plots for the type of inhibition (Fig. 5e). Like GD3-glycan, GT1b-glycan showed competitive inhibition toward GD3 binding (Fig. 5e), suggesting that GT1b binds to the same site as GD3. This is also consistent with the fact that GT1b attained a stable binding in the X-ray crystallographical analysis of the GT1b-Siglec-7 complex (2HRL)^22^. Other ganglioside glycans including GP1c, GQ1c, GT1c, GD1b, GD1a, and GM1a displayed non-competitive inhibition toward GD3 binding, and thus they also bound to the allosteric site other than the GD3 binding site. Notably, GT1a-glycan did not show any inhibitory effect on GD3-Siglec-7EcFc interaction, although it has diSia structure. We do not know the reason for that, but GT1a might have a specific conformational structure that prevents it from binding to the Siglec-7. Interestingly, GD1a- and GM1a-glycans were found to be strong non-competitive inhibitors toward GD3-Siglec-7EcFc interaction, irrespective of the presence of diSia structure in them (Table 2, Fig. 5d). On the other hand, in the ELISA-based binding experiments, no and slight binding toward GM1a and GD1a gangliosides, respectively, were observed (Supplementary Fig. 7b). These results indicate that, although these molecules should bind to the allosteric binding site other than GD3 binding site, they could not establish a stable binding toward GM1a and GD1a gangliosides. This is an interesting feature of these gangliosides, which remains to be explained. Taken all together, we can suggest that the GD3-Siglec-7 interaction is blocked by several ganglioside glycans, except GT1a, in a competitive and a non-competitive fashion, although the degree of effects is dependent on the ganglioside species.

**Table 2:** *K*_i_ values of synthetic inhibitors for GD3-Siglec-7 interaction.

Inhibitors	Inhibition type	*K*_*i*_
		μM as Sia
α2,8-diSia	Competitive	4.8 × 10^2^
α2,8-triSia	Non-competitive	5.4 × 10^2^
GM1a	Non-competitive	4.5 × 10^0^
GD1a	Non-competitive	1.1 × 10^1^
GD1b	Non-competitive	9.2 × 10^0^
GT1b	Competitive	1.3 × 10^1^
GQ1b	Competitive	1.0 × 10^3^
GT1c	Non-competitive	6.1 × 10^2^
GQ1c	Non-competitive	3.5 × 10^2^
GP1c	Non-competitive	1.3 × 10^2^
Poly-diSia^6^	Competitive	2.6 × 10^−2^
diSia-Dex^3^	Competitive	4.0 × 10^−1^

## Conclusions

**Figure 6:**
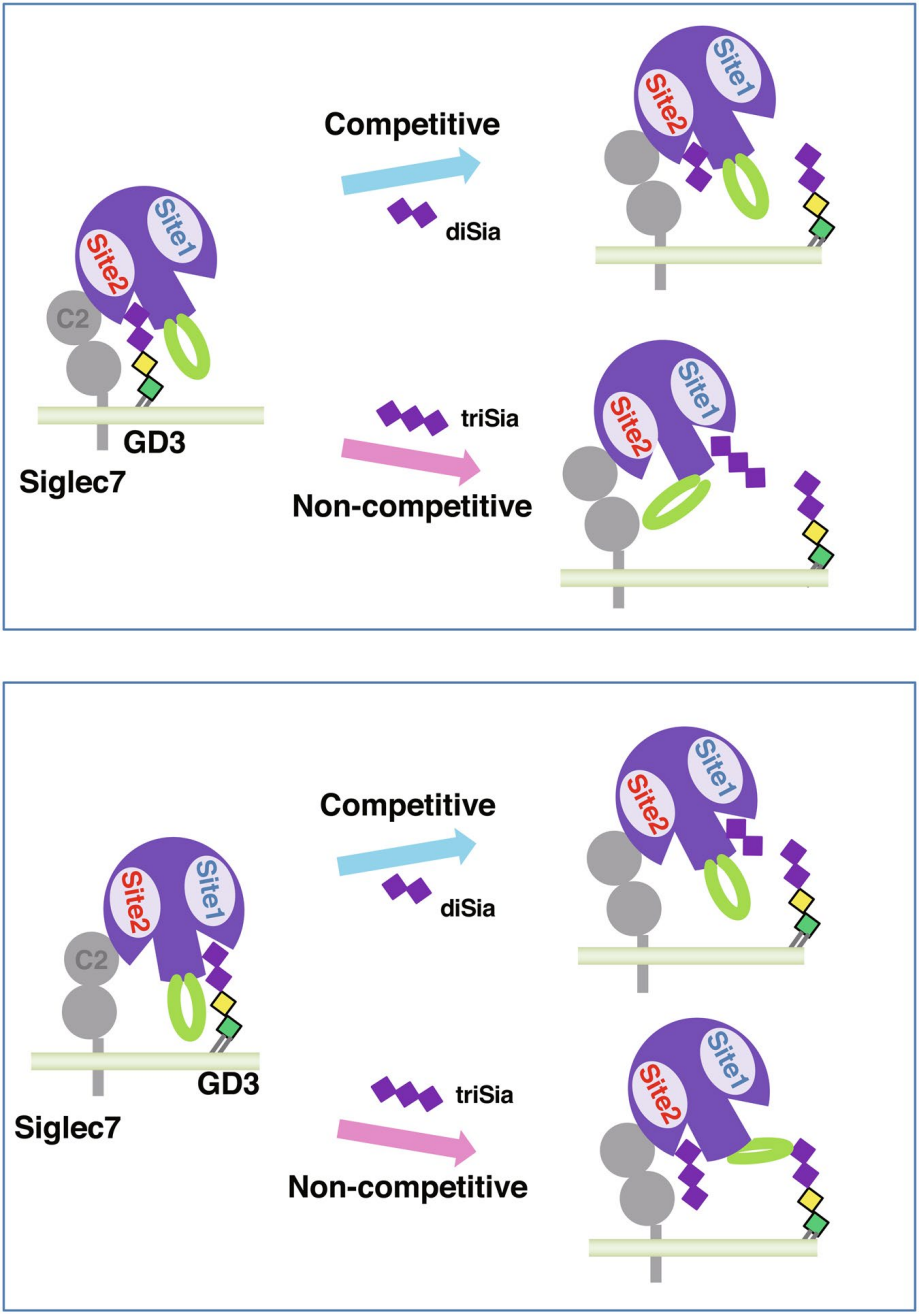
The two-binding-sites model of GD3-Siglec-7 binding. Site 1 is a conventional ligand-binding site, which binds to α2,8-diSia. Site 2 is a newly found Sia-binding site for α2,8-diSia. Not only α2,8-diSia, but also α2,8-triSia can bind to both sites of Siglec-7. Site 1 appears to work as an allosteric site as well as a ligand-binding site for site 2, and vice versa. (*Upper panel*) When site 2 is occupied by GD3, α2,8-diSia, and GT1b, glycan can competitively bind to site 2 to inhibit the GD3-site 2 binding. On the other hand, α2,8-triSia and several other ganglioside glycans non-competitively bind to site 1, leading to inhibition of the GD3-site 2 binding. In this case, site 1 works as an allosteric site for site 2, probably because site 1 binding induces the conformational change of the flexible C-C′ loop followed by the closure of site 2. (*Lower panel*) When Site 1 is occupied by GD3, α2,8-diSia and GT1b glycan can competitively bind to site 1 to inhibit the GD3-site 1 binding. On the other hand, α2,8-triSia and several ganglioside glycans non-competitively bind to site 2, leading to inhibition of the GD3-site 1 binding. In this case, site 2 works as an allosteric site for site 1, probably because site 2 binding induces the conformational change of the flexible C-C′ loop followed by the closure of the site 1. Sia, purple diamond; C-C′ loop, green; ◆◆, α2,8-dSia and GT1b-glycan; ◆◆◆, α2,8-triSia and several ganglioside glycans as described in the text.

Here we demonstrate the existence of a new Sia-binding site (site 2) in addition to the well-known primary Sia-binding site (site 1) in Siglec-7 based on the following lines of evidence. First, docking simulation using the apo and liganded forms of Siglec-7V predicts the importance of R67 in site 2 together with R124 in the conventional sialic acid-binding site 1. Second, the binding experiments using mutated Siglec-7EcFc variants and a variety of compounds demonstrated that site 2 is involved in the diSia and triSia binding in the same way as site 1. Third, by equilibration dialysis, Siglec-7 WT did not follow a single binding site-model, but rather may have at least two sites on the Siglec-7, suggesting the presence of a new sialic acid-binding site other than site 1. In addition, by equilibration dialysis and STD-NMR experiments, R124A appeared to close the new binding site toward diSia and triSia. Fourth, not only competitive inhibitors, but also non-competitive inhibitors toward diSia-Siglec-7EcFc interactions were found, indicating that a Sia-binding site other than site 1 is present to work as a regulatory site. Taken together, the results presented here are consistent with a model in which two binding sites contribute to glycan recognition by Siglec-7, with a conformational change of the flexible C-C′ loop mediating communication between these sites. Further studies will be required to elucidate structural details of the proposed allosteric mechanism (Fig. 6).

## Materials and Methods

### *In silico* molecular modelling of Siglec-7 and sialosides

#### Ligand preparation

For the docking experiment, several ligand molecules were built and optimised with either the SYBYL software (SYBYL, Tripos Inc., St Louis, Mo, USA, http://www.tripos.com/) or the molecular operating environment (MOE) molecular modelling package [Chemical Computing Group Inc., Montreal, Canada; Ryoka System, Inc., Tokyo, Japan]. The starting structures were optimised by using the random searching and minimisation tools in either SYBYL or MOE, respectively.

Specifically, the ligand structure Neu5Acα2-8Neu5Acα2-3Gal was constructed (Supplemental Fig. 2). Atomic partial charges were calculated using the AMBER99 option in MOE for estimating the distribution of charge over sigma bond networks. Then, the ligand coordinates were written into MOL2 format files.

#### Constructing the Siglec-7 V-set domain

The coordinates from the two (four) crystal structures of human Siglec-7 V-set domain (Siglec-7V), 1O7V.pdb (1.9 Å)^11^, and 2HRL.pdb (1.85 Å)^22^ were used as the protein models. For the starting structures of the docking simulations using Siglec-7V, we first modified the models to simplify the docking procedure. We first removed water molecules and co-crystallised ligand in 2HRL. Then, N-linked oligosaccharides attached to Asn105 in the crystal structures were removed. Each structure was modified by reconstructing the missing heavy atoms, adding hydrogens, and minimising to correct for bond or angle irregularities caused by the modifications described above, with AMBER force field.

#### Molecular docking simulations

The molecular docking simulations of Siglec-7V (residues 18–144, 1O7V; 25–144, 2HRL) were performed using the flexible ligand docking program DOCK 6.1 (http://dock.compbio.ucsf.edu/)^32^. The program DOCK 6.1 can be summarised as a search for geometrically allowed ligand-binding modes using several steps that include: describing the ligand and receptor cavity as sets of spheres, matching the sphere sets, orienting the ligand, and scoring the orientation. For each ligand, the molecular surface was calculated using a probe radius of 1.4 Å, and the spheres were generated with the DOCK program SPHGEN. SPHGEN output provided the spheres in clusters. Clusters were examined for the ligand, and the cluster covering the target-binding site was chosen. Some spheres were deleted to keep the target site confined to the cavity. The receptor and ligand were docked, allowing for ligand flexibility, and using the grid-based energy scoring option for minimisation after the initial placement in the site. The docked structures with each ligand were inspected using MOE both to optimise and to examine all energy calculations with the Merck force field (MMFF94x). The docking energy scores (U_*dock*_) indicated better interaction, representing the electrostatic and van der Waals interactions, between Siglec-7V and the disialylated glycans.

#### Materials

Restriction enzymes and Ex Taq polymerase were purchased from TaKaRa (Shiga, Japan). The Qiaex II kit was purchased from Qiagen (Hilden, Germany). GeneJuice transfection reagent was from Novagen (Darmstadt, Germany). Protein A-Sepharose and enhanced chemiluminescence western blotting detection reagents were purchased from GE Healthcare (Madison, WI, USA). Sialidase from *Vibrio cholerae*, was purchased from Sigma (St. Louis, MO, USA). Sialidase from *Arthrobacter ureafaciens* was obtained from Nacalai Co. (Kyoto, Japan). Polyvinylidene difluoride (PVDF) membrane (Immobilon P) and Ultra YM-10 were products from Millipore (Bedford, MA). GD3 (Neu5Ac*α*2 → 8Neu5A*α*2 → 3Gal*β*1 → 4Glc-Cer), and GM3 (Neu5A*α*2 → 3Gal*β*1 → 4Glc-Cer) were from Larodan Fine Chemicals (Malmo, Sweden). Dulbecco’s Modified Eagle’s Medium (DMEM) was obtained from Wako (Osaka, Japan). Serum-free medium (Cosmedium) was purchased from Cosmobio (Tokyo Japan). *Clostridium perfringens* exo-sialidase, and bovine serum albumin (BSA) were purchased from Sigma-Aldrich (Missouri, USA). Peroxidase (POD)-labelled anti-rabbit IgG was obtained from Cell Signalling (Danvers, USA). POD-labelled anti-mouse IgG + IgM and anti-human IgG + IgM + IgA were purchased from American Qualex (CA, USA).

#### Plasmid construction

The pcDNA3.1 plasmids encoding the full length of Siglec-7 and the extracellular domain of Siglec-7 fused with the human IgG1 Fc region; pcDNA3.1-Siglec-7EcFc and pcDNA3.1-Siglec-7FL were used^3^. The pcDNA3.1-Siglec-7EcFc (R67A), -Siglec-7EcFc (R67K), -Siglec-7EcFc (R124A), and -Siglec-7EcFc (R124K) were prepared using PCR with the primer pairs as shown in Supplementary Table 2 (50 ng primer), with PfuDNA polymerase (1U) and pcDNA3.1-Siglec-7EcFc (WT) as a template. After DpnI digestion (Takara, Shiga, Japan) of PCR products at 37 °C for 1 h, the digested PCR products were transformed, and the plasmids were prepared in each colony. All the pcDNA3.1-Sigelc7 plasmids were confirmed by the deoxynucleotide chain termination-based method.

#### Purification of Siglec-7-Fc fusion proteins

The Siglec-7-Fc chimeric protein was produced by stably expressing the established CHO cells using the G418 selection reagent, which was after transfection with pcDNA3.1-Sigelc7EcFc using the GeneJuice transfection reagent. The stably expressed CHO cells were incubated in DMEM with 10% foetal bovine serum, and then changed to a serum-free medium (Cosmedium 005, Comobio, Japan). After incubating for 14 d, the cell supernatants were harvested and the Fc chimera protein was purified from culture supernatants using protein A-Sepharose chromatography. Siglec-7EcFc protein was concentrated with YM-10 membrane and resuspended in TBS (50 mM Tris-HCl pH 8.0, 140 mM NaCl). Protein concentrations were determined densitometrically using western blotting with human IgG as a standard.

The conformation of the protein was analysed by the ELISA using three different antibodies that can be used for intact protein. The Siglec7EcFc (0–40 ng) was immobilized via precoated protein A onto the plastic wells and washed with PBS. Then three different antibodies, 7.5^25^, 7.7^25^ and polyclonal anti-Siglec7 (R&D systems, Minneapolis, USA) that are able to use in flowcytometry analysis, were incubated at 37 °C for 2 h. After washing, peroxidase-conjugated secondary antibodies were incubated at 37 °C for 2 h. After washing, then 100 µL of 0.05% o-phenylenediamine in 0.1 M Tris-HCl (pH 6.8) containing 0.006% H_2_O_2_ was added and incubated at room temperature. The absorbance was then measured at 490 nm (Bio-Rad, Hercules, CA) after the addition of 100 µL of 2 N sulphuric acid.

#### ELISA analysis

For ganglioside binding assay, 96-well plates (Nunc, Roskilde, Denmark) were coated with 50 μL/well of 0–6.0 µM ganglioside in ethanol and left to air dry overnight or at 37 °C for 2 h. The wells were washed three times with TBS and blocked with 1% BSA (w/v) in TBS. Siglec-7-Fc fusion protein (64 pmol/mL) was pre-complexed with a 1/1000 dilution of horseradish peroxidase-conjugated goat anti-human IgG + M + A (American Qualex) for 30 min at room temperature, and 50 μL of reaction mixture was added to each well. After 2 h incubation at room temperature, the wells were washed extensively, then 100 µL of 0.05% o-phenylenediamine in 0.1 M Tris-HCl (pH 6.8) containing 0.006% H_2_O_2_ was added and incubated at room temperature. The absorbance was then measured at 490 nm (Bio-Rad, Hercules, CA) after the addition of 100 µL of 2 N sulphuric acid. For measurement of the inhibition toward GD3-Siglec-7 interaction, inhibitors (0–200 μM for octyl-α2,8-mono/di/tri/teraSia, 0–50 μM for tetradecyl-ganglioside glycan), Siglec-7, and peroxidase conjugated anti-human IgG + IgM + IgA were pre-incubated at 25 °C for 30 min, and the reaction mixture with or without inhibitor was used as described above. All experiments were performed three times.

#### STD-NMR

Recombinant human Siglec-7 V-set domain and its mutants (R67A, R124A, R67K) were prepared using the pCold-PDI vector as previously described with modifications^33^. DNA fragments encoding human Siglec-7 V-set domain (residues 18–144) and the mutants were sub-cloned into the pCold-PDI vector. The plasmids were transformed into *Escherichia coli* Origami B (DE3) (Novagen), and the cells were grown at 37 °C in LB medium. After induction with isopropyl β-D-thiogalactoside (0.5 mM), the cells were cultured at 15 °C for further 16 h. Then the cells were harvested and sonicated in 20 mM Tris-HCl (pH 8.0), and containing 0.1 M NaCl and 0.1 mM EDTA. After centrifugation, the supernatant containing (His)_6_-PDI-fused protein was collected and applied to a Ni sepharose column (GE healthcare) equilibrated with 20 mM Tris-HCl (pH 8.0), and containing 0.1 M NaCl. After washing with 20 mM Tris-HCl (pH 8.0) containing 0.1 M NaCl and 20 mM imidazole, the protein was eluted with 20 mM Tris-HCl (pH 8.0) containing 0.1 M NaCl and 500 mM imidazole. The (His)_6_-PDI-fused protein was extensively dialysed against 50 mM Tris-HCl (pH 8.0) containing 0.1 M NaCl, 5 mM reduced glutathione, 0.5 mM oxidised glutathione, and 10% glycerol. The protein was then digested with TEV protease to remove the (His)_6_-PDI tag. The digested proteins were passed through the Ni sepharose column and the final purification was performed with a Hiroad Superdex 75 pg size exclusion column (GE healthcare) equilibrated with 20 mM Tris-HCl (pH 8.0) containing 0.1 M NaCl.

NMR spectra were collected using the DRX-600 (600 MHz) spectrometer (Bruker; Billerica, MA, US) equipped with a TXI-probe. The probe temperature was set at 283 K. Siglec-7 V-set domains (WT, R124A, R67A) were prepared with 10 mM phosphate buffer including 150 mM NaCl in D_2_O (D: 99%). The final protein concentration before addition of the ligand was 55 μM (pH 7.9). The 5.5 mM diSia and triSia solutions (pH 7.9) in PBS were gradually added into the protein solution to achieve the protein:ligand molar ratios of 1:0, 1:1, 1:2, 1:5, 1:10, and 1:20; the ^1^H-NMR spectra were obtained with the WATERGATE pulse sequence in each protein:ligand ratio. Data were collected for 1024 scans by suppressing the residual HDO signal to 3-9-19 pulse train. Saturation Transfer Difference (STD) NMR experiments were performed at the protein:ligand ratio of 1:20. The protein signal was saturated on resonance at 0.15 ppm and off resonance at 40 ppm with a cascade of 60 selective Gaussian shaped pulses of 50 ms duration and 3 s saturation in total. The WATERGATE pulse sequence was used to suppress the residual HDO signal, and a spin-lock filter with a strength of 8 kHz and a duration of 10 ms was applied to suppress protein background signal. All STD NMR experiments were collected for 1024 scans with an inter pulse delay of 100 μs. The Sias signal assignments have been previously described by Jennings *et al*.^34^.

#### Equilibrium dialysis experiment

Equilibrium dialysis was performed according to the protocol of RED Devise (Thermo Scientific). Rhodamine green conjugated Neu5Acα2,8Neu5Acα2,3Gal^35^ at 1 × 10^−9^~1.6 ×^−8^ M and Siglec-7EcFc and its mutants at 2.5 × 10^−10^ M were used for the analyses. The analyses were performed three times. The values are expressed as mean ± SD.

#### Chemical synthesis of sialoglycan

The α-(2,8)-di, tri, and tetra Sias were prepared as previously reported^31^. Ganglioside epitopes (GQ1c, GT1c, GQ1b, GT1b, GD1b, GT1a, GD1a, GM1a) were prepared according to the previously reported method^36^ for the synthesis of a GP1c ganglioside epitope. The synthetic materials did not form micelles under the conditions in this study.

#### Flow cytometric analysis

RAW cell surface staining using a reaction mixture of Siglec-7-Ec-Fc (64 pmol/mL) and anti-human IgG-Alexa 488 (1/1000) was measured using a flow cytometer (Gallios, Beckman Coulter), and the data obtained was analysed with the Kaluza software (Beckman Coulter). For glycan analysis, cells were incubated with 10–20 μg/ml of FITC-conjugated RCA1 and SNA (Seikagaku-co, Tokyo, Japan), anti-diSia antibody (S2-566)^27^, or anti-triSia antibody (A2B5)^27^. After washing the cells, anti-Sia antibody stained cells were incubated with anti-mouse IgM-Alexa488 (1/500). After washing, all cells were measured using a flow cytometer.

## Supplementary information

Supplementary information.

## References

[1] Crocker PR, Paulson JC, Varki A (2007). Siglecs and their roles in the immune system. Nat. Rev. Immunol..

[2] Büll C, Heise T, Adema GJ, Boltje TJ (2016). Sialic acid mimetics to target the sialic acid-siglec axis. Trends Biochem. Sci..

[3] Yamaguchi S (2017). Chemical synthesis and evaluation of a disialic acid-containing dextran polymer as an inhibitor for the interaction between siglec 7 and its ligand. Chembiochem.

[4] Hudak JE, Canham SM, Bertozzi CR (2014). Glycocalyx engineering reveals a siglec-based mechanism for NK cell immunoevasion. Nat. Chem. Biol..

[5] Prescher H (2017). Design, synthesis, and biological evaluation of small, high-affinity siglec-7 ligands: toward novel inhibitors of cancer immune evasion. J. Med. Chem..

[6] Ohira S (2017). Synthesis of end-functionalized glycopolymers containing α(2,8) disialic acids via π-allyl nickel catalyzed coordinating polymerization and their interaction with Siglec-7. Chem. Commun. (Camb).

[7] Xiao H, Woods EC, Vukojicic P, Bertozzi CR (2016). Precision glycocalyx editing as a strategy for cancer immunotherapy. Proc. Natl. Acad. Sci. USA.

[8] Chen WC (2010). *In vivo* targeting of B-cell lymphoma with glycan ligands of CD22. Blood.

[9] Nicoll G (1999). Identification and characterization of a novel siglec, siglec-7, expressed by human natural killer cells and monocytes. J. Biol. Chem..

[10] Angata T, Varki A (2000). Siglec-7: a sialic acid-binding lectin of the immunoglobulin superfamily. Glycobiology.

[11] Alphey M, Attrill H, Crocker P, van Aalten D (2003). High resolution crystal structures of siglec-7. Insights into ligand specificity in the Siglec family. J. Biol. Chem..

[12] Yamaji T, Teranishi T, Alphey MS, Crocker PR, Hashimoto Y (2002). A small region of the natural killer cell receptor, Siglec-7, is responsible for its preferred binding to alpha 2,8-disialyl and branched alpha 2,6-sialyl residues. A comparison with Siglec-9. J. Biol. Chem..

[13] Kawasaki Y (2010). Ganglioside DSGb5, preferred ligand for Siglec-7, inhibits NK cell cytotoxicity against renal cell carcinoma cells. Glycobiology.

[14] Rosenstock P, Horstkorte R, Gnanapragassam VS, Harth J, Kielstein H (2017). Siglec-7 expression is reduced on a natural killer (NK) cell subset of obese humans. Immunol. Res..

[15] Varchetta S (2016). Lack of siglec-7 expression identifies a dysfunctional natural killer cell subset associated with liver inflammation and fibrosis in chronic HCV infection. Gut..

[16] Brunetta E (2009). The decreased expression of siglec-7 represents an early marker of dysfunctional natural killer-cell subsets associated with high levels of HIV-1 viremia. Blood.

[17] Shao JY (2016). Siglec-7 defines a highly functional natural killer cell subset and inhibits cell-mediated activities. Scand. J. Immunol..

[18] Varchetta S (2012). Engagement of siglec-7 receptor induces a pro-inflammatory response selectively in monocytes. PLoS One.

[19] Ikehara Y, Ikehara SK, Paulson JC (2004). Negative regulation of T cell receptor signaling by siglec-7 (p70/AIRM) and siglec-9. J. Biol. Chem..

[20] Dimasi N, Moretta A, Moretta L, Biassoni R, Mariuzza RA (2004). Structure of the saccharide-binding domain of the human natural killer cell inhibitory receptor p75/AIRM1. Acta Crystallogr. D. Biol. Crystallogr..

[21] Attrill H (2006). The structure of siglec-7 in complex with sialosides: leads for rational structure-based inhibitor design. Biochem. J..

[22] Attrill H (2006). Siglec-7 undergoes a major conformational change when complexed with the alpha(2,8)-disialylganglioside GT1b. J. Biol. Chem..

[23] Ito A, Handa K, Withers DA, Satoh M, Hakomori S (2001). Binding specificity of siglec7 to disialogangliosides of renal cell carcinoma: possible role of disialogangliosides in tumor progression. FEBS Lett..

[24] Rapoport E, Mikhalyov I, Zhang J, Crocker P, Bovin N (2003). Ganglioside binding pattern of CD33-related siglecs. Bioorg. Med. Chem. Lett..

[25] Nicoll G (2003). Ganglioside GD3 expression on target cells can modulate NK cell cytotoxicity via siglec-7-dependent and -independent mechanisms. Eur. J. Immunol.

[26] Sato C (1995). Characterization of the antigenic specificity of four different anti-(alpha 2–>8-linked polysialic acid) antibodies using lipid-conjugated oligo/polysialic acids. J. Biol. Chem..

[27] Sato C, Kitajima K (2013). Disialic, oligosialic and polysialic acids: distribution, functions and related disease. J. of Biochem..

[28] Mayer M (2001). Group epitope mapping by saturation transfer difference NMR to identify segments of a ligand in direct contact with a protein receptor. J. Am. Chem. Soc..

[29] Hashimoto N (2019). The ceramide moiety of disialoganglioside (GD3) is essential for GD3 recognition by the sialic acid-binding lectin SIGLEC7 on the cell surface. J. Biol. Chem..

[30] Tanaka H, Nishiura Y, Takahashi T (2009). Stereoselective synthesis of alpha(2,9) di- to tetrasialic acids, using a 5,4-N,O-carbonyl protected thiosialoside. J. Org. Chem..

[31] Tanaka H, Nishiura Y, Takahashi T (2006). Stereoselective synthesis of oligo-alpha-(2,8)-sialic acids. J. Am. Chem. Soc..

[32] Moustakas DT (2006). Development and validation of a modular, extensible docking program: Dock 5. J. Comput. Aided Mol. Des..

[33] Subedi GP (2012). Overproduction of anti-Tn antibody MLS128 single-chain Fv fragment in *Escherichia coli* cytoplasm using a novel pCold-PDI vector. Protein Expr. Purif..

[34] Michon F, Brisson JR, Jennings HJ (1987). Conformational differences between linear alpha (2-8)-linked homosialooligosaccharides and the epitope of the group B meningococcal polysaccharide. Biochemistry.

[35] Sato C, Yamakawa N, Kitajima K (2010). Measurement of glycan-based interactions by frontal affinity chromatography and surface plasmon resonance. Methods Enzymol..

[36] Tanaka H, Nishiura Y, Takahashi T (2008). An efficient convergent synthesis of GP1c ganglioside epitope. J. Am. Chem. Soc..

