# Supplementary material for: Discovery of a new sialic acid binding region that regulates Siglec-7
Source: Sci Rep. 2020 May 26;10:8647. doi: 10.1038/s41598-020-64887-4 (PMC7250851; doi:10.1038/s41598-020-64887-4)

Supporting information

Discovery of a new sialic acid binding region that regulates Siglec-7

Nao Yamakawa[1,2,3,#], Yu Yasuda[1,2,#], Atsushi Yoshimura[1,2], Ami Goshima[1,2], Paul R. Crocker[4], Gérard Vergoten[3], Yuji Nishiura[5], Takashi Takahashi[6], Shinya Hanashima[7,8], Kana Matsumoto[7], Yoshiki Yamaguchi[7,9], Hiroshi Tanaka[5], Ken Kitajima[1,2] and Chihiro Sato[1,2,*]

[1]Biocience and Biotechnology Center, Nagoya University, Nagoya, 464-8601, Japan,
[2]Department of Bioagricultural Sciences, Nagoya University, Nagoya, 464-8601, Japan,
[3]Université de Lille, CNRS, UMR 8576, Unité de Glycobiologie Structurale et Fonctionnelle, Lille 59000, France
[4]Division of Cell Signalling and Immunology, School of Life Sciences, University of Dundee, Dundee DD1 5EH, UK
[5]Department of Chemical Science and Engineering, School of Materials and Chemical Technology, Tokyo Institute of Technology, 2-12-1-H-101, Ookayama, Meguro, Tokyo 152-8552, Japan
[6]Department of pharmacy, Yokohama University of Pharmacy, 601, Matana-cho, Totsuka-ku, Yokohama, Kanagawa 245-0066, Japan
[7]Structural Glycobiology Team, RIKEN Global Research Cluster, 2-1 Hirosawa, Wako, Saitama 351-0198, Japan
[8]Present address: Graduate School of Science, Osaka University, 1-1 Machikaneyama, Toyonaka, Osaka 560-0043, Japan
[9] Present address: Faculty of Pharmaceutical Sciences, Tohoku Medical and Pharmaceutical University, 4-4-1 Komatsushima, Aobaku, Sendai, Miyagi 981-8558, Japan

#These authors contributed equally to this work.

[*]Correspondence to C. S. (chi@agr.nagoya-u.ac.jp)

LEGENDS FOR SUPPLEMENTARY FIGURES

Supplementary Figure 1. Crystal structures of Siglec-7. (a) Typical crystal structure of Siglec-7 (1O7V). The helix and strands are shown. Loop B-C and loop C-C' are circled in the dotted line. (b) Superimposed structures of Siglec-7 using six crystal structures, 1NKO, 1O7V, 1O7S, 2DF3, 2G5R, and 2HRL. 1NKO, 1O7V, 1O7S are the apo form and 2DF3, 2G5R, and 2HRL are the liganded form. Liganded form was prepared using MOE systems. (c) Magnification of the well-known conventional Sia-binding site. The side chains of the important amino acid residues, including R124 are shown.

Supplementary Figure 2. Ligand structure used for docking analysis. The ligand used in this study, a Neu5Acα2,8Neu5Acα2,3-Galβ1-CH_3 structure is shown.

Supplementary Figure 3. Purification and protein folding check of Siglec-7EcFc. (a) Silver staining of purified Siglec-7. Wild type Siglec-7EcFc (WT), mutated Siglec-7EcFc R67A, and R124A sample purified from culture medium derived from CHO cells stably secreting Siglec-7EcFc after transfection of pcDNA3.1-Siglec-7EcFc plasmids and selection with antibiotics, using protein A-Sepharose. The purified proteins were detected by silver staining. (b) Siglec-7WT and mutated Siglec-7EcFc R67A, R67K, R92A, R92K, R124A, and R124K purified by the same procedure were detected using POD-labeled anti-human Ig (lower panel) and anti-human Fc antibody followed by POD-labeled anti-mouse Ig (upper panel) for the detection of Fc, before (-) and after (+) treatment with sialidase. (c) The non-treated Siglec-7EcFc samples were evaluated for conformation by Siglec-7 specific antibodies using ELISA. Siglec-7EcFc was incubated onto the Protein A-pre-immobilized plate and washed. The conformation of the Siglec-7EcFc was evaluated by two anti-Siglec-7 monoclonal antibodies (7.7. and 7.5.)[37] and one polyclonal antibody toward Siglec-7 (poly) that can be used for the flowcytometric analysis. The bound antibodies were quantitated by colorimetric analysis (A490). (d) ELISA analysis of asialo-Siglec-7EcFc and Siglec-7EcFc. The di/oligoSia-PE were coated onto the plastic well plate and blocked. After washing, the premixed sialidase-treated or untreated Siglec-7EcFc with anti-human Ig antibody labelled with POD were incubated. The binding of Siglec-7EcFc was measured after colorimetric development.

Supplementary Figure 4. Sia epitope analyses of RAW mouse macrophage cells by flow cytometry. RAW cells were incubated with RCA1 lectin for asialoGal epitope, SNA lectin for monoSia epitope, monoclonal antibody S2-566 for diSia epitope, and monoclonal antibody A2B5 for triSia epitope. The incubated cells were stained with Alexa 488-labelled secondary antibodies. The stained cells were analysed using flow cytometer. Left panel shows the flow cytometric data.

Supplementary Figure 5. Equilibrium dialysis experiments of Siglec-7. Equilibrium dialysis was performed using 1 x 10^{-9} ~ 1.6 x 10^{-8} M of rhodamine green-conjugated Neu5Acα2,8Neu5Acα2,3Gal[35] as ligand in both the buffer and the sample compartments. In the sample compartment, 2.5 x 10^{-10} M of Siglec-7EcFc (WT) and its mutants were added. After 4 h at 37°C, the fluorescence was measured using a fluorometer (fluoroMAX3) (Ex. 503 nm, Em. 530 nm). The analyses were performed in triplicate. Binding profiles and the Scatchard plots are shown in the left and right panels, respectively, for each of Siglec-7 (WT), (R67A), and (R124A). r, amount of the bound ligand per Siglec-7 (mol/mol); [S], concentration of free ligand; n, number of ligand-binding site per Siglec-7 molecule. Calculated values for K_A and n from these data are summarised in Supplementary Table 3.

Supplementary Figure 6. STD-NMR. ^{1}H-NMR spectra of Siglec-7 WT and its mutants R124A and R67A titrated with the diSia or triSia ligand in 0, 1, 2, 5, 10, and 20 mole equivalents. The aromatic/amide region (7.7–6.2 ppm) and the aliphatic region (1.3–0 ppm) were arrayed. The ligand molar ratio-dependent changes of some protein signals indicate ligand-binding and/or conformational change. The initial protein concentration was 55 μM in 10 mM PBS (pH 7.9). The probe temperature was set at 10 °C. Dotted lines and dotted squares in red indicate the significant shift. Dotted lines and dotted squares in yellow indicate the weak shift.

Supplementary Figure 7. Inhibition toward GD3-Siglec-7 interaction. (a) The plate was coated with GD3 (50 nM). The complexed mixture with Siglec-7 and anti-human IgG+IgM+IgA were incubated with or without synthetic ganglioside glycans as described in Material and Method. Then we added the mixed solution onto the GD3-immoblised plate and the interaction toward GD3 was measured at A490. The GD3 binding without

the synthetic compounds as inhibitors were set to 1.0. The experiments were performed three times and SE are shown. Upper panel, c-series; Middle panel, b-series; Lower panel, a-series. (b) The binding of Siglec-7EcFc to gangliosides GM1a, GD1a, GD1b, and GD3 (0 - 1500 nM) was analysed using ELISA. The ELISA values (A490) are measured as described under Materials and Methods.

Supplementary Table 1. Docking results for the low-binding-energy complexes of a disialylated glycan to 2G5R and 2DF3 models using DOCK 6.1. Combinations of amino acid residues and glycan residues that are involved in hydrogen bonding and salt bridge are listed. Sia 1, Sia 2, and Gal stand for residues in Sia2-Sia1-Gal, corresponding to Neu5Acα2-8Neu5Acα2-3Gal-Me. The number of intermolecular hydrogen bond and salt bridge, the binding energy (U_{dock}) are also shown in the list. These are top-hit conformations.

Model protein	2G5R	2G5R	2DF3	2DF3
**Docking site**	**Site 1**	**Site2**	**Site 1**	**Site 2**
**U_{dock} (kcal/mol)**	**-258**	**-169**	**n.d.[1]**	**-204**
**The number of internal hydrogen bond and salt bridge**	**8**	**7**		**9**
**Amino acid and glycan residues involved in the hydrogen bond and salt bridge**				
**S21**	Sia2			
**Y26**	Sia2			
**Y64**				
**R67**		Sia2		Sia2
**G69**		Sia2		
**N70**		Sia1		Sia2
**D71**		Gal		
**S73**				
**K75**				
**E90**		Sia2		
**R92**				
**D117**				
**R120**				Sia2
**R124**	Sia2			
**E126**				
**G128**				
**N129**	Sia2			
**K131**				
**N133**	Sia1			Gal
**K135**	Sia1			Sia1

[1]n.d. not detected.

Supplementary Table 2 Primers used in this study for point mutation

Primer Name	Sequence
R67K sense	5'-GTTCATGGCTACTGGTTCAAGGCAGGGA-3'
R67K antisense	5'-CTTATATCATTCCCTGCCTTGAACCAGT-3'
R92K sense	5'-CAGGAGGAAACTAAGGACCGATTCCACC-3'
R92K antisense	5'-GGTGGAATCGGTCCTTAGTTTCCTCCT-3'
R124K sense	5'-GGGAGATACTTCTTTAAGATTGGAGAAAGGAAAT-3'
R124K antisense	5'-ATTTCCTTTCTCCATCTTAAAGAAGTATCTCCC-3'

Supplementary Table 3. Summary of the equilibrium dialysis experiments

K_A and n values were calculated from the Scatchard plots of r (amount of the bound ligand per Siglec-7) and [S] (concentration of free ligand).

	Siglec-7 (WT)		Siglec-7 (R67A)	
**Trial**	**K_A (M^{-1})**	**n**	**K_A (M^{-1})**	**n**
**1**	8.5 $x10^8$	3.4	2.0 $x10^8$	3.0
**2**	5.1 $x10^8$	4.4	7.0 $x10^8$	2.9
**3**	4.5 $x10^8$	5.3	2.0 $x10^8$	3.0
**All (1, 2, 3)[a]**	5.5 $x10^8$	4.2	2.6 $x10^8$	2.8
**Av. (1-3)[b]**	6.0 $x10^8$ ($\pm$2.1 $x10^8$)	4.3 ($\pm$0.81)	3.8 $x10^8$ ($\pm$2.1 $x10^8$)	3.0 ($\pm$0.080)

[a]All the data obtained in three experiments were used for the plots as shown in Supplementary Fig. 5.

[b]The K_A and n values were obtained from three Scatchard plots of r/[S] and r. The data are shown as mean ± SD from three independent experiments.

Supplementary Fig. 1

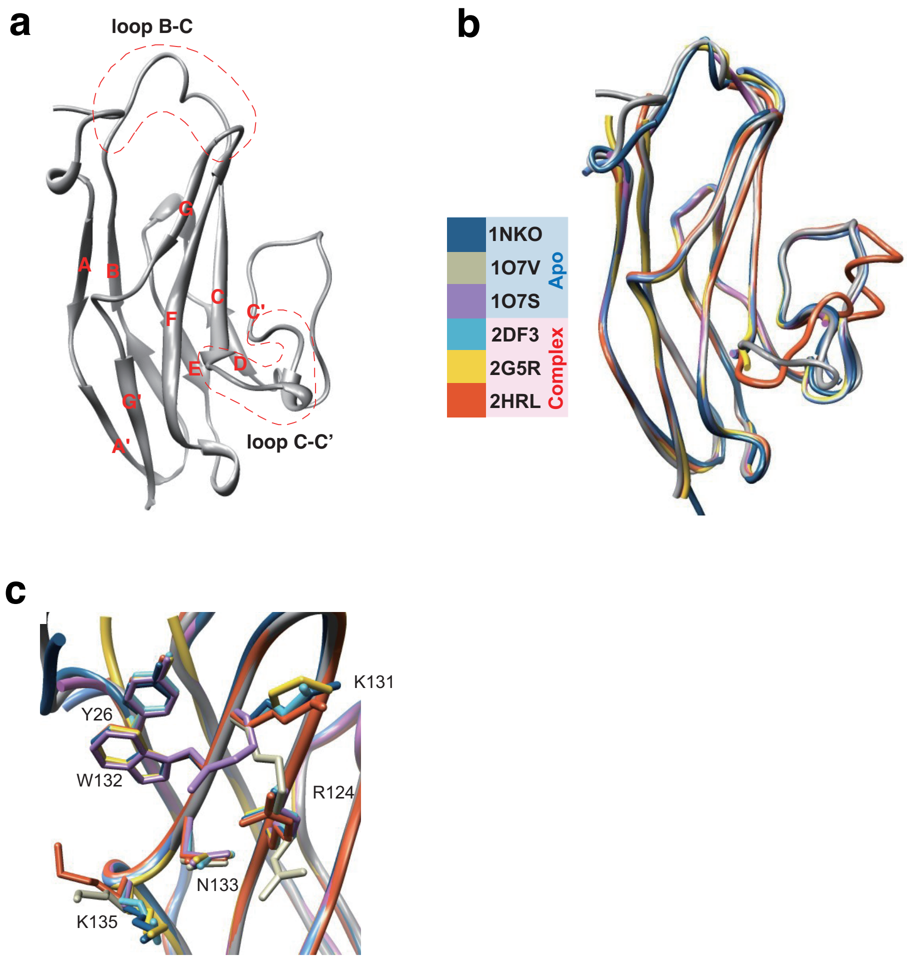

Supplementary Fig. 2

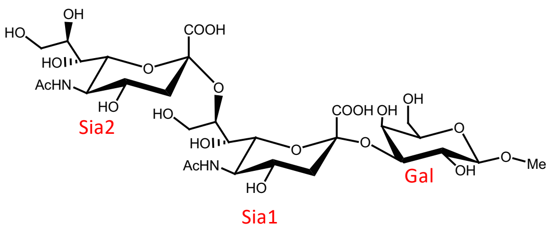

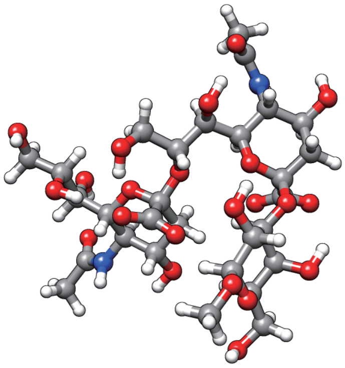

methyl β-glycoside of Neu5Acα2-8Neu5Acα2-3Gal

Supplementary Fig. 3

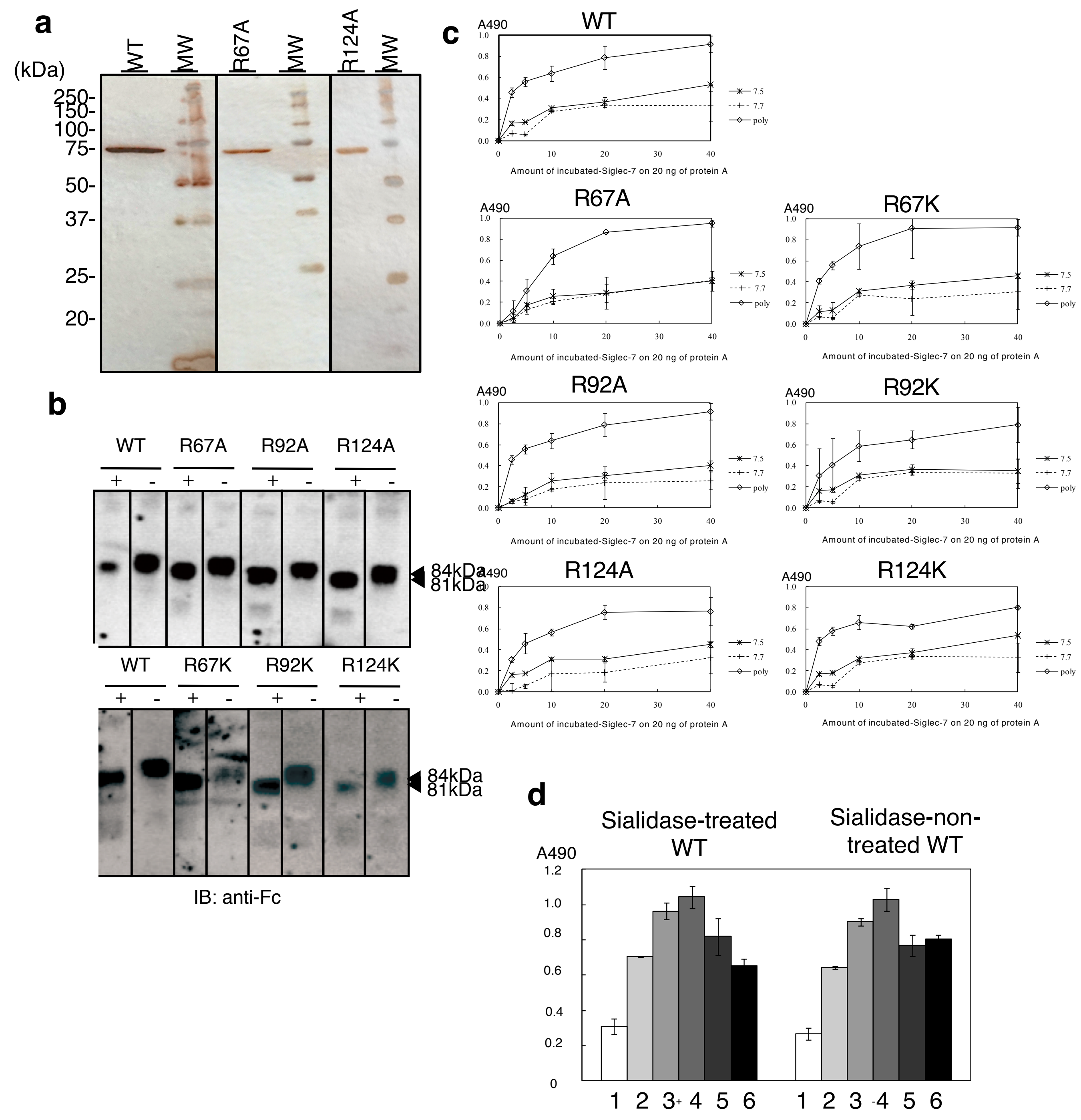

Supplementary Fig. 4

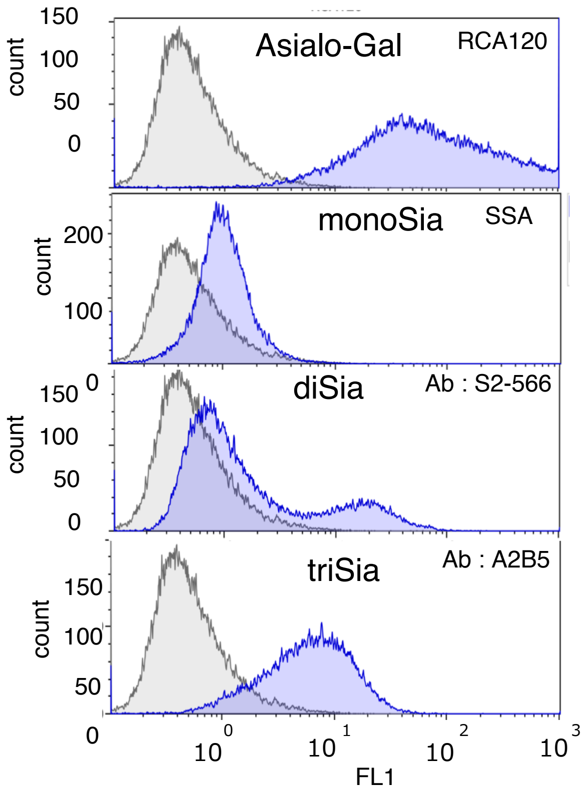

Supplementary Fig. 5

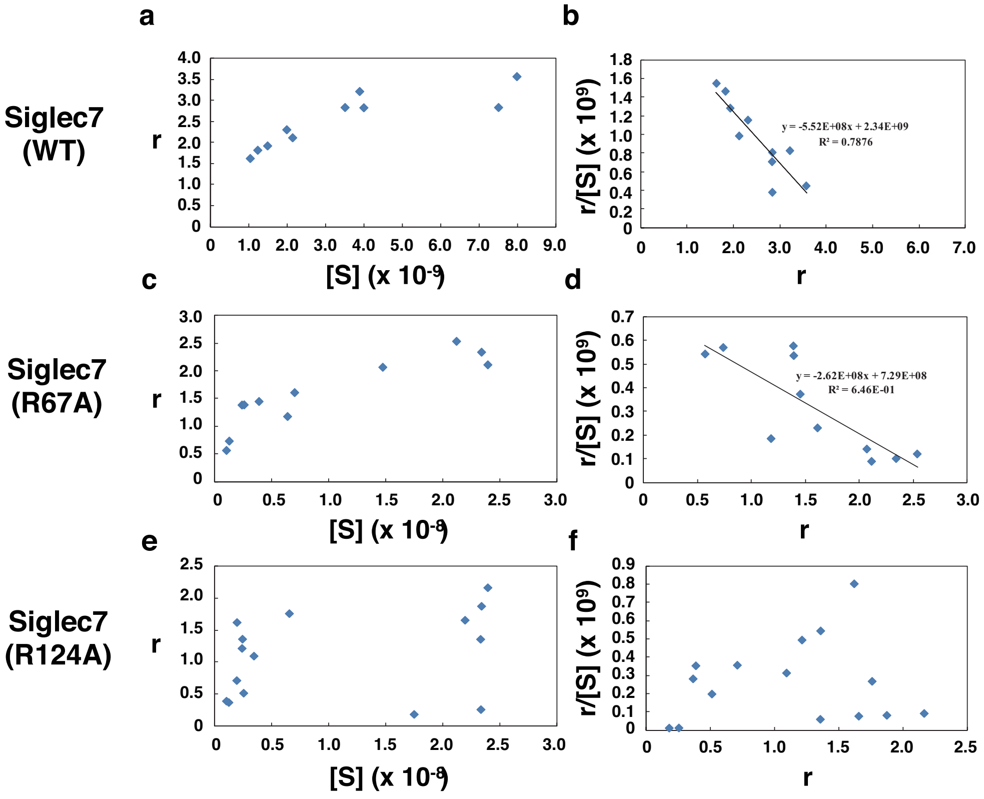

Supplementary Fig. 6

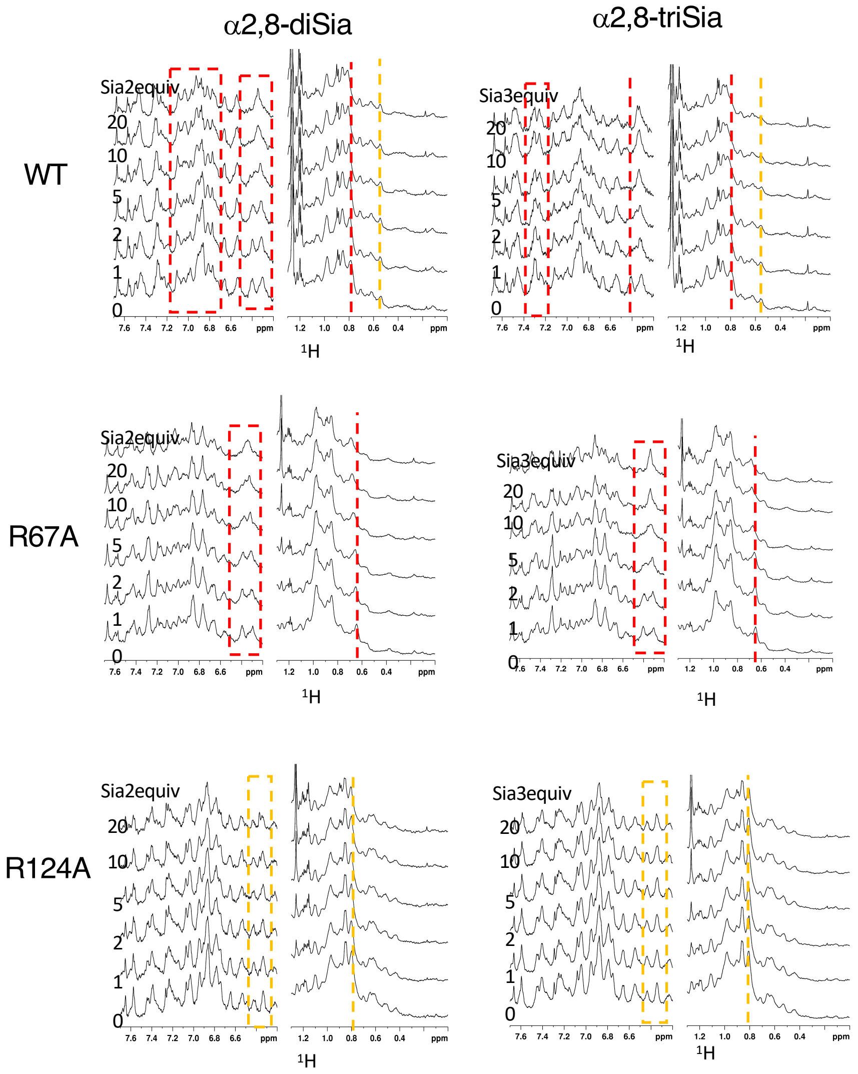

Supplementary Fig. 7

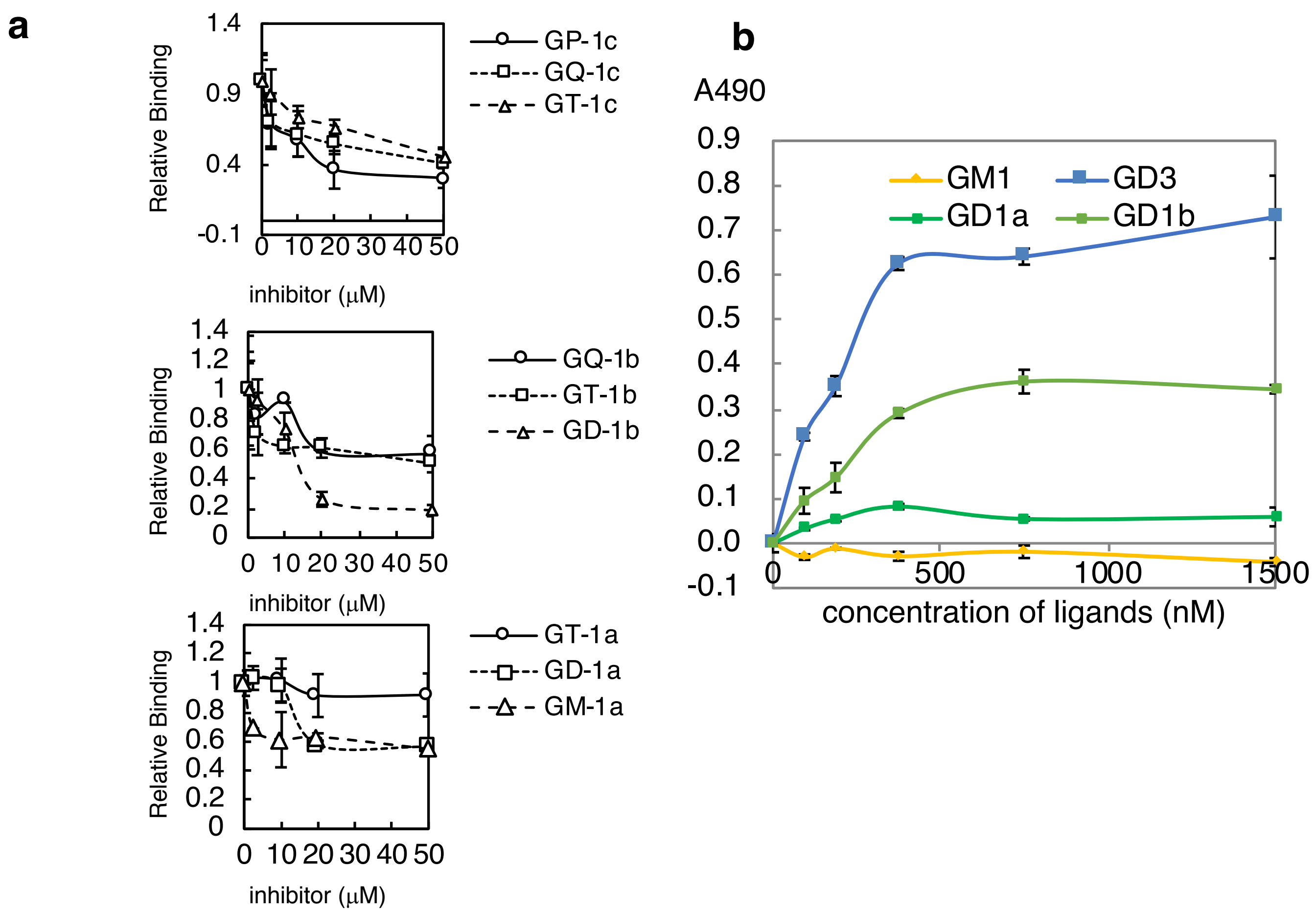

Supplement: Supplementary file 1 — Supplementary information. [file 41598_2020_64887_MOESM1_ESM.pdf]
